# Exploring public perception and utilization of medication home delivery services in the United Arab Emirates: a cross-sectional study

**DOI:** 10.1186/s12913-024-11770-x

**Published:** 2024-11-04

**Authors:** Feras Jirjees, Amna M Othman, Mohanad Odeh, Hala AlObaidi, Zelal Kharaba, Hawraa Adhari, Menna Elshenawy, Fatima Almaazmi, Yahya H Dallal Bashi, Mamoon A Aldeyab, Ahmad Al-Azayzih, Rana Abu Farha, Nermin Eissa, Karem H Alzoubi

**Affiliations:** 1 College of Pharmacy, University of Sharjah, PO Box 27272, Sharjah, United Arab Emirates; 2 Department of Clinical Pharmacy and Pharmaceutical Sciences, Faculty of Pharmaceutical Sciences, the Hashemite University, Zarqa, Jordan; 3 College of Humanities, City University Ajman, Ajman, United Arab Emirates; 4 School of Pharmacy, Queens University Belfast, Belfast, UK; 5 Faculty of Medical Sciences, Newcastle University, Newcastle, UK; 6 Department of Pharmacy, School of Applied Sciences, University of Huddersfield, Huddersfield, UK; 7 Faculty of Pharmacy, Applied Science Private University, Amman, Jordan; 8 College of Health Sciences, Abu Dhabi University, PO Box 59911, Abu Dhabi, United Arab Emirates

**Keywords:** Medication home delivery, United Arab Emirates, Community pharmacy, Pharmacist, Mobile Application, People perceptions

## Abstract

**Introduction:**

The Medication Home Delivery (MHD) service from community pharmacies involves the safe and efficient delivery of pharmaceuticals (prescription and non-prescription medications), and health products directly to the patient’s/consumer’s home. There are several issues encountered by the users of the MHD service that have an impact on their satisfaction with the service. The study aimed to assess the public’s perceptions of the MHD service in the United Arab Emirates (UAE), their willingness to utilize it, and the practical use of the service.

**Method:**

A cross-sectional exploratory study was conducted in the UAE using a validated online survey. The inclusion criteria were adults residing in the UAE. Statistical analysis was performed to identify the association between the variables, the service use, and the level of service efficiency.

**Results:**

A total of 556 participants filled out the survey, with 69.4% of them using the service. The majority of participants were females (75.9%) and aged less than 40 years old (71.6%). Three variables showed a statistically significant association with the use of the MHD service (*P* < 0.05): the participant’s educational level, their medical/health background, and the frequency of visits to community pharmacies. The most common issues raised were receiving the wrong order, delay in delivery, and paying delivery fees. Most participants agreed that the MHD service reduces the risk of exposure during pandemics, serves the elderly, serves disabled people, makes it more comfortable for parents with children at home, and reduces overcrowding in health facilities, as well as the MHD service making pharmacy services more efficient.

**Conclusion:**

The study indicated positive perceptions among the public in the UAE towards the MHD service. However, there was a concern that this service may diminish the communication between pharmacists and patients, which potentially minimizes the amount of information received by patients regarding their treatments.

**Supplementary Information:**

The online version contains supplementary material available at 10.1186/s12913-024-11770-x.

## Introduction

Community pharmacies are recognized as the most accessible and utilized frontline healthcare facilities worldwide due to the healthcare requirements of the population, the nature of the pharmaceutical service, and its easy accessibility. In addition, the role of pharmacists has greatly evolved primarily due to the development and expansion of the pharmacy profession from a dispensing-oriented to a patient-oriented profession by providing patient care, health guidance, and medication counselling [1, 2]. Moreover, the rapid developments in communication technologies provide community pharmacies an additional tool for providing pharmaceutical services to people in their homes [3].

One of the pharmacy-based services provided by community pharmacies is medication home delivery (MHD) which refers to delivering pharmaceutical products directly to patients’ homes or preferred locations without having to visit the pharmacy [4, 5]. It involves safe and efficient delivery of prescribed medications, non-prescribed medications, and other health products by licensed pharmacies, under specified guidelines, to avoid errors while providing this service [6]. This MHD service is designed to enhance convenience and accessibility for patients; particularly those with limited mobility, acute health problems, or patients who require regular medication refills. Furthermore, this service includes providing information to patients on their medications. The main benefit of this service is reducing overcrowding and unnecessary visits to pharmacies and accordingly reducing the load on pharmacies. Moreover, it allows the patients to receive their medications without interruption during critical times such as acute diseases and pandemics. On the other hand, it may contribute to medication errors if not correctly implemented through a structured system with close monitoring and follow-up. In fact, according to the National Pharmacy Association, the MHD service may result in around 9% of errors [7]. In this MHD service, the most common errors are medicines delivered to the wrong patients, wrong labeling on the delivered package, inappropriate patient counseling, and suboptimal medication therapy management [8].

To ensure an efficient MHD service, medications must be labelled, stored, and transported appropriately. It is also necessary for pharmacists to maintain communication with the patients through proficient telecommunication tools to avoid dispensing errors. Moreover, pharmacists need to address all patient inquiries and questions. They must also assist patients to help them understand medication and treatment related information, and to identify the needs of some groups of patients such as children, pregnant women, and the elderly. A study by Kavanagh et al. found that patients had concerns about medication safety and delivery time and emphasized on the significance of clear communication and easy access to information. Furthermore, there are concerns about the potential for medication misuse and the need for secure delivery methods and authentication procedures [9]. Several studies reported that the MHD service provided by the community pharmacies was an efficient pharmaceutical service according to consumers [10, 11]. Thus, patients can get their medications directly delivered to their preferred locations, often more conveniently than other alternatives [4, 5].

The MHD service provided by community pharmacies was introduced decades ago in many countries, and it has gradually grown. In recent years, the MHD service has become increasingly popular, and is being offered by many pharmacies, and e-commerce platforms. In addition, the demand increased markedly in light of the COVID-19 pandemic, where medication delivery became a popular alternative to traditional in-person pharmacy visits [3, 11, 12].

The United Arab Emirates (UAE) is one of the first countries in the Middle East region to adopt this service and provide it according to defined regulations [13]. The rules of the MHD service were officially approved by the Ministry of Health and Prevention, and other health authorities in 2020, in which the community pharmacies can deliver medications around the UAE after completing all requirements from the health authority [7]. Currently, the MHD service is offered by most community pharmacies in the country. In addition, many online pharmacy and delivery service companies provide the MHD service. Usually, this service is provided for consumers in short time (hours).

Since little information is available about consumers’ perspectives on the service in the UAE, this study was conducted to investigate the public’s perceptions (users and non-users) of the MHD service in the UAE, their willingness to utilize it, and their practical use of the service.

## Methods

### Study design and participants

A survey-based cross-sectional study was carried out over two months between the mid of October to the mid of December 2022. The study was conducted through an online survey created on the Google form platform. Recruitment efforts were made through various social media platforms (WhatsApp and Facebook) and online forums, inviting participants to take part in the online survey assessing their perceptions of the MHD service in the UAE. Participants were given a comprehensive introduction about the objectives of this study, and they were required to review and consent to a participation agreement prior to commencing the survey.

Inclusion criteria comprised residents of the UAE, both male and female, who were at least 18 years of age. However, the study excluded pharmacists, and individuals working in the field of pharmacy. The questionnaire did not include any personal information for the participants.

### Questionnaire development and validation

A questionnaire was adopted from a previous research study conducted in Jordan. This questionnaire was translated to Arabic following the forward-backward translation method [11]. The contents of the questionnaire were modified to be more applicable to the UAE residents including examples of typical mobile applications, and delivery companies of pharmaceutical products. Furthermore, additional questions were added to the survey. The questionnaire was bilingual: Arabic and English. To ensure the content validity of the questionnaire, three independent academic pharmacists with a panel of experts in pharmacy practice and six individuals with various educational backgrounds (non-pharmacy background) were given the questionnaire to be evaluated. Minor changes were made according to the feedback.

The optimised questionnaire consisted of five sections; (1) contains items of sociodemographic characteristics, (2) evaluates public awareness and their support for the MHD service, (3) asks for the participants’ opinions about the difference between getting medication through the MHD service or in pharmacy (face-to-face), (4) asks participants to evaluate the benefits of utilizing the MHD service, and (5) examines how well the participant perceived the drawbacks of using this service, the limitations, the chance of developing medication errors, the efficacy of counseling, and medication accuracy by using the MHD service. The Likert Scale was used to document responses for some questions in the last three sections. The questionnaire took around 15 min to be completed.

### Sample size calculation

A snowball sampling technique was used in this study. The Raosoft^®^ software sample size calculator was used to estimate the sample size [14]. The calculation was performed for the sample size required for a population of any size. An expected frequency of 50% was used in the absence of similar studies in the country, a 95% confidence interval, and a margin of error of 5%. The minimum sample size required was of 384 participants.

### Ethical approval and consent to participate

This study was approved by the research ethics committee at the University of Sharjah, UAE (REC-22-05-S). All methods were performed by the relevant guidelines and regulations or declaration of Helsinki.

### Data analysis

Data from the online survey were downloaded into a secure database and analyzed using the 28th version of the statistical package for Social Science (SPSS^®^). Descriptive statistics, including the mean ± standard deviation (SD) and percentages were used for continuous and categorical variables to calculate all variables of interest. Statistical analysis (Chi-squared test) was conducted to identify the association between using the service and demographic variables. It was also used to identify the associations between using the service and its efficiency level. Furthermore, to determine the strength of association Cramer’s phi (φc) coefficient, where values > 0.15 were considered strong association, > 0.1 and > 0.05 moderate and weak association, respectively [15]. The figures were produced by Microsoft Excel.

To enhance comprehension of the factors affecting the differentiation between MHD users and non-users, we performed a multivariable logistic regression analysis. The dependent variable was defined as the utilization of the service, while the predictors encompassed the studied variables. Adjusted Odds ratios (OR), along with their respective P values, were computed, alongside 95% confidence intervals (CI) to provide a comprehensive insight into the relationships.

## Results

### Sociodemographic characteristics of the participants and relation with the MHD service

A total of 556 participants filled out the survey, with 386 participants (69.4%) who requested at least an order from the community pharmacy over the last 12 months. Conversely, 170 participants (30.6%) did not make any purchases remotely or online from the pharmacy.

Most participants were females (75.9%) and less than 40 years old (71.6%). Also, more than half of the participants (57.6%) had children and approximately half of the participants (49.8%) had a medical or health background. Furthermore, over a quarter of the participants (26.1%) had chronic diseases. In addition, all participants who used the MHD service were still visiting the community pharmacy; however, their visits were more frequent compared with the non-users group. In addition, a large proportion of participants appeared to have educational degrees.

Table 1 shows the association between variables and the use of the MHD service. Only four variables showed a statistically significant association, the education level (*p* = 0.006), participants with a medical/health background (*p* = 0.011), participants with chronic diseases (*p* = 0.020), and the frequency of visits to community pharmacy (*p* = 0.0015). The strongest association was seen with the education level (φc = 0.16).

Following multivariable analysis, the adjusted odds ratio and p values revealed that education, participants with medical/health background and the frequency of visits to community pharmacy retained statistical impact for association of being MHD users. As determined by the multivariable model, Individuals with a bachelor’s degree exhibited a 3.1 times higher likelihood of being MHD users compared to non-educated participants (OR = 3.1, 95% CI = 1.2–7.6, *p* = 0.02). This effect was followed by a 2.01 times likelihood for postgraduates (OR = 2.01, 95% CI = 1.1–8.9, *p* = 0.04), while the differences among those with Primary – high school or Diploma degree and non-educated appeared to be not significant (OR = 0.76, 95% CI = 0.48–1.3, *p* = 0.3 and OR = 1.6, 95% CI = 0.7–3.5, *p* = 0.22 respectively).

Individuals with a medical background exhibit a 2.3 times higher likelihood of being MHD users compared to those without a medical background (OR = 2.3, 95% CI = 1.5–3.49, *p* < 0.001). Furthermore, individuals who reported weekly visits to the pharmacy show a 5.3-fold increase in the tendency to be MHD users compared to those with fewer visits (OR = 5.3, 95% CI = 2.2–13.1, *p* < 0.001). Finally, the status of chronic disease for the individuals failed to maintain significant impact upon corrections in the multivariable model. See Table (1).

**Table 1:** Sociodemographic characteristics of the study participants and association with using the medication home delivery service (*n*=556)

Variables	n (%)	Users of the MHD service n (%)		*P* Value^a^	Cramer’s phi (φc)	*P* Value^a^	Adjusted OR^b^	95% CI	
	Total n=556	No n=170 (30.6%)	Yes n=386 (69.4%)					Lower	Upper
**Age**									
≤ 40years	398 (71.6%)	130 (76.5%)	268 (69.4%)	0.300	0.10	**Reference**			
>40 years	158 (28.4%)	40 (23.5%)	118 (30.6%)			0.15	0.25	0.04	1.6
**Gender**									
40Male	133 (23.9%)	45 (26.5%)	88 (22.8%)	0.290	0.04	**Reference**			
Female	422 (75.9%)	125 (73.5%)	298 (77.2%)			0.06	1.6	0.9	2.7
**Education level**						**0.015**			
Non-formal education	5 (0.9%)	1 (0.6%)	4 (1.0%)	**0.006**	0.16	Reference			
Primary – high school	151 (27.4%)	50 (29.4%)	102 (26.4%)			0.3	0.76	0.48	1.3
Diploma degree	55 (10.0%)	7 (4.1%)	48 (12.4%)			0.22	1.6	0.7	3.5
Bachelor’s degree	272 (49.3%)	97 (57.1%)	178 (46.1%)			**0.02**	3.1	1.2	7.6
Postgraduate degree	69 (12.5%)	15 (8.8%)	54 (14.0%)			**0.04**	2.01	1.1	8.9
**Marital status**						0.23			
Divorced or widowed	24 (4.2%)	8 (4.7%)	16 (4.1%)	0.470	0.05	Reference			
Single	320 (57.8%)	104 (61.2%)	216 (56.0%)			0.29	5.1	0.2	109.4
Married	212 (38.0%)	58 (34.1%)	154 (39.9%)			0.18	2.7	0.40	18.4
**Participants having children**									
No	236 (42.4%)	66 (38.8%)	170 (44.0%)	0.210	0.05	Reference			
Yes	320 (57.6%)	104 (61.2%)	216 (56.0%)			0. 38	1.6	0.82	2.4
**Participants living with elderly people (over 65 years)**									
No	431 (77.5%)	131 (77.1%)	300 (77.7%)	0.710	0.03	Reference			
Yes	125 (22.5%)	39 (22.9%)	86 (22.3%)			**0.06**	0.78	0.37	1.6
**Participants with medical/health background**									
No	279 (50.2%)	99 (58.2%)	180 (46.6%)			Reference			
Yes	277 (49.8%)	71 (41.8%)	206 (53.4%)	**0.011**	0.11	** <0.001**	2.3	1.5	3.49
**Participants with chronic diseases**									
No	411 (73.9%)	137 (80.1%)	274 (71.0%)	**0.020**	0.10	Reference			
Yes	145 (26.1%)	33 (19.4%)	112 (29.0%)			0.48	0.8	0.46	1.44
**Visit to community pharmacy**						**0.002**			
< three months	273 (49.1%)	98 (57.6%)	175 (45.3%)	**0.002**	0.15	Reference			
Weekly	62 (11.2%)	8 (4.7%)	54 (14.0%)			**<0.001**	5.3	2.2	13.1
Monthly	221 (39.7%)	64 (37.6%)	157 (40.7%)			0.055	1.6	0.9	2.7

^a^Bold font illustrated statistically significance at the level of <0.05

^b^Adjustment based on multivariable logistic regression

### Communication methods, purposes of using the MHD service, and problems encountered by the participants

The phone was the most common tool used by the participants who used the MHD service to contact the pharmacy as reported by 56.7% of the participants, then the mobile applications (such as WhatsApp/Messenger) that was used by 56.2% of the participants. Furthermore, 38.1% of the participants contacted the pharmacy through ordering company applications.

More than half (63.2%) of the participants who used the MHD service order medicines that do not require a prescription, while less than half of them (43.0%) asked to fill a prescription, and almost 6.2% of them use the MHD service to order both prescribed and non-prescribed medications.

Half of the participants (*n* = 193) who used the MHD service reported that there is at least one problem, and 80 participants (20.7%) reported more than one problem. The most common problems or issues raised by the participants were due to the late delivery (28.5%), and paying the delivery fees (26.7%). In addition, receiving wrong orders, and no answers to their questions were also reported from 8.3% to 8.0% of the participants, respectively. Table 2 shows communication approaches, purposes of using the MHD service, and problems faced among participants who used the service.

**Table 2:** Approaches and purposes of using the pharmacy delivery service, and problems encountered by the participants who used the service (*n*=386)

	**n (%)**
**Approaches to contacting the pharmacy**	
By phone	219 (56.7%)
By mobile application	217 (56.2%)
Using ordering company applications	147 (38.1%)
**Purpose of using the MHD service**	
Order medicines that do not require a prescription	244 (63.2%)
Order personal care products	170 (44.0%)
Order supplements/nutritional products	171 (44.3%)
Request to fill a prescription	166 (43.0%)
Order medical equipment	110 (28.5%)
**Problems encountered by participants**	
Late delivery	110 (28.5%)
Paying delivery fee	103 (26.7%)
Receiving wrong order	32 (8.3%)
No answer to my question	31 (8.0%)
The medication required a prescription	27 (7.0%)
I did not receive my order	19 (4.9%)
Receiving damaged order	18 (4.7%)

### Participants’ opinions about the MHD service

Table 3 presents participants’ opinions about the MHD service. Large proportions of participants who have either used or never used the MHD service (77.7% and 73.5%, respectively) were contented with principals/presence of this service in the UAE and confirmed the efficiency of the MHD service (85.4% and 71.2%, respectively). However, among participants who had never used the service before, 7.1% were not contented with the service compared to only 1.6% of the users group. In addition, they believed that the service does not improve pharmacy services by 11.2% for the non-users group compared to only 4.7% for the users group.

**Table 3:** Participants’ opinions about the medication home delivery service

	The use of MHD service (*n*=556)				Used service (n=386)			
	Yes *n*=386 (69.4%)	No *n*=170 (30.6%)	*P*	Cramér's phi (φc)	With problem *n*=193(50.0%)	No problem *n*=193(50.0%)	*P*	Cramér's phi (φc)
	n (%)				n (%)			
**The support toward introducing medication home delivery service in community pharmacies**								
Supportive	300 (77.7%)	125 (73.5%)	**0.030**	0.14	142 (73.6%)	158 (81.9%)	0.13	0.10
Neutral	80 (20.7%)	33 (19.4%)			48 (24.9%)	32 (16.6%)		
Unsupportive	6 (1.6%)	12 (7.1%)			3 (1.6%)	3 (1.6%)		
**Providingmedications home delivery service makes pharmacy services more efficient**								
Yes	331 (85.4%)	121 (71.2%)	**<0.001**	0.18	157 (81.3%)	174 (90.2%)	**0.04**	0.13
No	18 (4.7%)	19 (11.2%)			12 (6.2%)	6 (3.1%)		
I do not know	37 (9.7%)	30 (17.6%)			24 (12.4%)	13 (6.7%)		

The participants who used the MHD service demonstrated statistically significant contentment with the service, and confirmed the efficiency of the service with *P* = 0.03 and < 0.001, respectively. Statistically, the strength of satisfaction with the efficiency of the MHD service was strong (φc = 0.18), and it was moderate (φc = 0.14) for supporting the service in community pharmacies.

Opinions of the participants who used the MHD service and encountered at least one problem were also different. The participants who did not report any issues were more contented with the MHD service compared to those who did report problems (81.9% vs. 73.6%). They also confirmed that the MHD make pharmacy services more efficient with 90.2% of them versus 81.3% for those who did not encounter issues. The latter was shown to be a strong and statically significant association (φc = 0.13 and *p* = 0.04, respectively).

Table 4 presents the participants’ opinions for using the MHD service compared with visits to the community pharmacy to get some pharmaceutical services. Less than half of the participants (43.8%) who used the MHD service preferred to visit the pharmacy to get non-prescribed (over-the-counter) medications (versus the non-users’ group with 52.9%), and almost two third of them (63.7%) to collect medication/pharmaceutical products during the COVID-19 pandemic (versus the non-users’ group with 60.6%). However, participants who used the MHD service were less preferred to collect prescribed medications (11.9%), and ask questions and get information related to medicine (14.5%) versus the non-users’ group (24.1% and 27.1%, respectively).

**Table 4:** Participants’ opinions to use the MHD service, or visiting the community pharmacy related to some pharmaceutical services

	Using the MHD service		Visiting community pharmacy		No difference between the two methods	
	Used of the MHD service n (%)					
	Yes (*n*=386)	No (*n*=170)	Yes (*n*=386)	No (*n*=170)	Yes (*n*=386)	No (*n*=170)
Ask questions and get information (advising) about my medications	56 (14.5%)	41 (24.1%)	242 (62.7%)	108 (63.5%)	88 (22.8%)	21 (12.4%)
Get non-prescribed (over-the-counter) medication	169 (43.8%)	90 (52.9%)	90 (23.3%)	53 (31.2%)	127 (32.9%)	27 (15.9%)
Collect prescribed medication	46 (11.9%)	46 (27.1%)	236 (61.1%)	95 (55.9%)	104 (26.9%)	29 (17.1%)
Collect medication/pharmaceutical product during the pandemic	246 (63.7%)	103 (60.6%)	81 (21.0%)	55 (32.4%)	59 (15.3%)	12 (7.1%)

### Benefits and drawbacks of using the MHD service

**Fig. 1:**
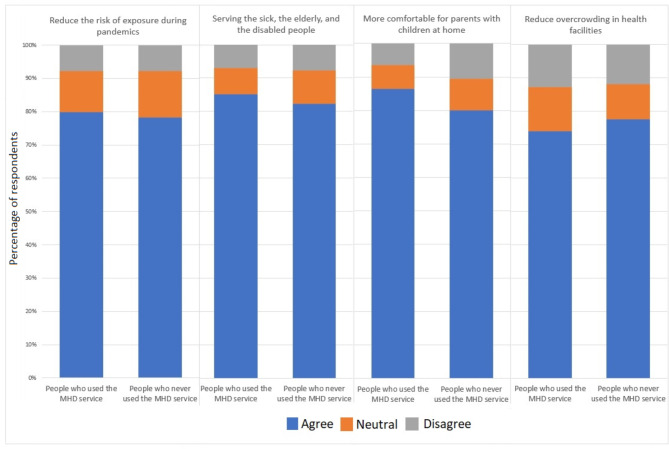
The participant’s opinions about the benefits of the MHD service (*n* = 556)

Figure 1 shows opinions on the benefits of the MHD service among participants who used and never used the MHD service. More than 78% of all participants agreed about all the benefits of the MHD service: it reduces the risk of exposure during pandemics, serves the sick, elderly, and disabled people (85.2% of users group vs. 82.4% non-users group), and more comfortable for parents with children at home (86.5% of users group and 80.0% non-users group), and reduces overcrowding in health facilities (75.1% of users group vs. 77.6% non-users group).

**Fig. 2:**
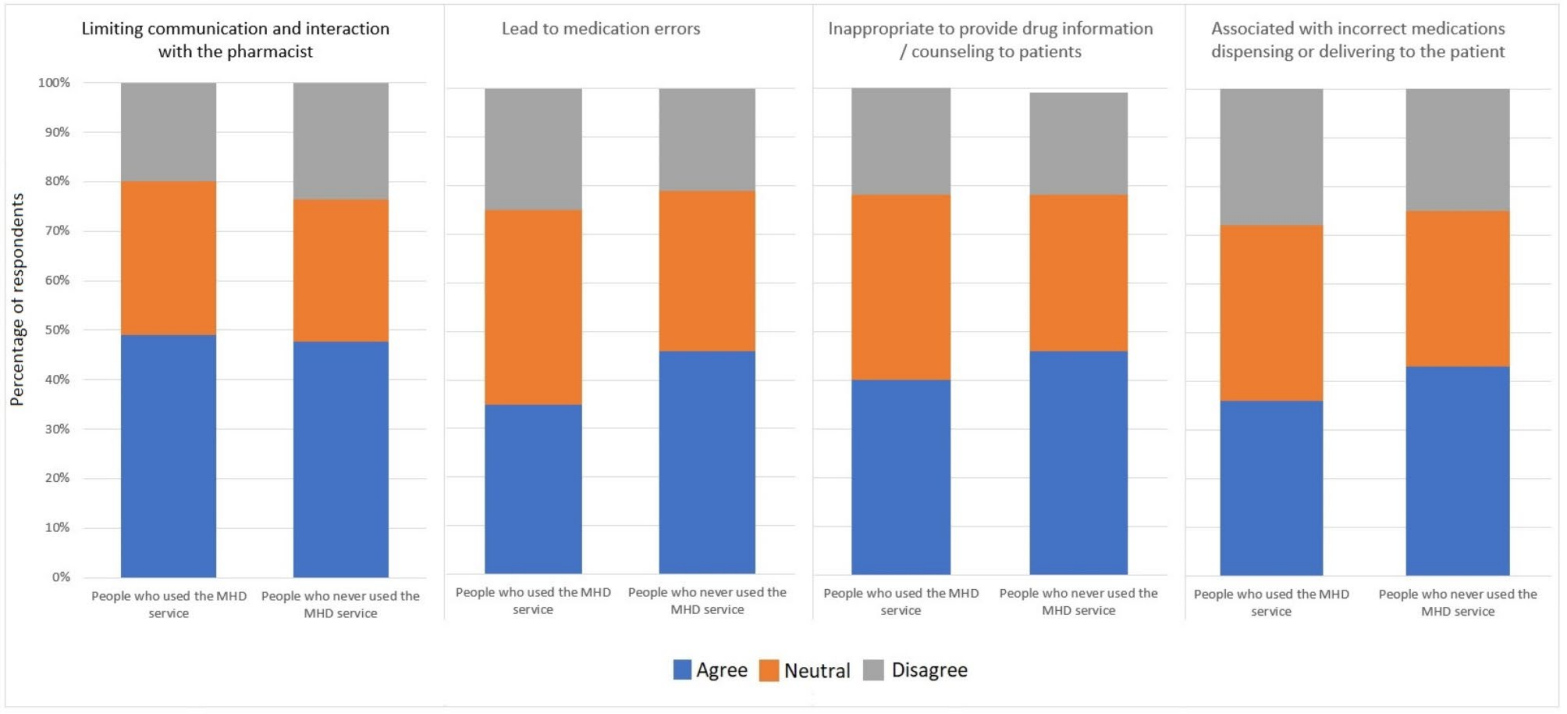
The participants’ opinions on the drawbacks of the MHD service (*n* = 556)

Figure 2 shows the drawbacks of the MHD service from the participants. There were no differences between the participants related to drawbacks of the MHD service. Around 50% of the participants agreed that the MHD service may limit the communication and interaction with the pharmacist. Furthermore, between 35 and 45% of the participants agreed that the MHD service might lead to medication errors, inappropriate drug information/counseling, and associated with incorrect medications dispensing or delivering to the patient.

## Discussion

Patients and customers using the MHD service and filling prescriptions from home became a common worldwide practice [3, 4, 9, 11, 16, 17]. The COVID-19 pandemic is one of the factors that has greatly increased the demand for this service, and made more people aware of it to reduce transmission of the virus [18]. A moderate increase in patients’ ability to adapt this service was reported [3, 11, 16]. However, further investigation of strategies to facilitate patient use of this service is needed. More countries worldwide have introduced the MHD service after the pandemic [4, 16, 17, 19, 20]. In the UAE, the regulations were issued shortly prior the pandemic, and the service was highly used during the pandemic time and onward [3].

In the UAE, there are several factors that make the MHD service successful. First, the presence of the polices regulating this service to ensure it is offered in a safe and efficient way [13]. The second is the infrastructure of the telecommunication technologies where the country has an efficient telecommunication network that is essential to offer remote healthcare services. In addition, the number of active internet users was reported to be 99% of the total population in 2020, in which almost all people in the UAE are using smart phone devices [2]. This developed infrastructure, telecommunications and internet network in the UAE have enabled community pharmacies to offer efficient telepharmacy services. Most community pharmacies offer the MHD service, and the vast majority of them are providing it for free. In addition, the high prevalence of chronic diseases in the UAE is associated with an increase in the use of the telepharmacy services [18, 21–23].

The findings of the current study indicated that the MHD service was accepted by most participants from the two groups who used or never used the pharmacy MHD service; however, some users encountered problems when ordering a product through this service. Furthermore, the majority of the participants reported that this service is useful to particular groups of people such as the elderly, people with disabilities, and families with children. Besides, the participants agreed that the service has benefits for the community during the pandemic period, and agreed that providing the service will make pharmacy services more efficient.

Although the number of young adult participants was over two-thirds of the participants, there was no significant association between the use or not use of the MHD service and the age of the participants. However, young adults are more oriented towards online shopping, including health products such as cosmetics and skin care products [24, 25], where they can get it online at less prices, which are not normally available in pharmacies. On the other hand, most old people preferred the traditional way of dealing with the pharmacy through physical visits and face-to-face contact with the pharmacist. However, more people of all ages preferred to use the MHD service in response to the COVID-19 pandemic due to associated precautionary measures during that period and to reduce/stop the transmission of the virus [3, 11, 25–27]. Moreover, chronic diseases, which are more prevalent in the elderly, led to more demand for prescription filling [17]. However, refilling chronic disease medications through the MHD service typically necessitates a prescription, a requirement often absent when visiting pharmacies in numerous countries worldwide [28, 29].

The multivariable analysis confirms that higher education, medical background, and the frequency of pharmacy visits are essential elements in shaping the likelihood of using the MHD service, ruling out the other potential factors such as the presence of chronic diseases. This suggests that high levels of education may enhance individuals’ awareness, understanding, or perceived need for the MHD service. The higher probability of using the MHD service was observed among postgraduates, than that of bachelor’s degree holders. This reinforces the idea that advanced education provides individuals with the service-related knowledge. This points to a possible gap in knowledge regarding this service among those with relatively low educational attainment, which could be an area for targeting public health interventions. In addition, the finding likely reflects the medical knowledge and practices that these individuals have, leading to greater understanding and trust in the usefulness of the MHD service. It also suggests that individuals with medical training or medical work experience are more likely to use this service, which possibly due to a higher degree of confidence in using medicines. A study conducted in Lebanon by Abu-Farha et al. research group revealed a significant association between educational level and MHD service use among the participants [30]. However, a study in Saudia Arabia reported that participants with a high school education have higher levels of satisfaction than other groups [31]. Conversely, another study conducted in Saudia Arabia indicated that there was no significant relationship between education levels and satisfaction level with the MHD service among the participants [32].

Finally, the frequency of visits to community pharmacies emerged as the most potent predictor of MHD use, with weekly visitors being over five times more likely to use the service. This finding is particularly compelling because the needs of this group of patients/consumers to the MHD service, as it may save time and effort for these people to collect their pharmaceutical products. In addition, it highlights the role of community pharmacies in enhancing individuals’ awareness of the service available through announcements. This could place the MHD service as a pivotal player in community pharmacies strategies aimed at increasing the adoption of the MHD service. This finding is comparable to the finding of a study conducted in Jordan showed a significant association between frequency of visiting a community pharmacy per month and use the MHD service among the participants [11]. Conversely, a study conducted in Lebanon found no significant association between individuals who visited a community pharmacy more frequently per month and those who visited less frequently [30].

Interestingly, the chronic disease status did not retain statistical significance in predicting the MHD service use after adjustments in the multivariable model. This outcome might initially seem counterintuitive, as individuals with chronic diseases would typically be expected to have a greater demand for health monitoring and management tools. This finding is similar to the results of a study conducted in Jordan [11]. However, the chronic disease status has a significant association with the use of the MHD service among study participants in Lebanon [30]. Though, this could indicate that other factors, such as education or medical background, may override the influence of chronic disease status, or that chronic disease alone is insufficient to drive the MHD service use without these other factors being present.

There were many barriers encountered by the participants. The reported obstacles can be classified under three categories: barriers related to the delivery service, barriers related to pharmaceutical services including communication with the pharmacist, and barriers related to a policy which mainly regulates the eligibility to deliver the prescribed medication. For the first barrier, as per the participants, the main issue was late delivery of an order, which affected the quality of the service. This would be a problem if the patient takes medications for acute problems and chronic illnesses, which may have an effect on medications taken regularly. Similar results were reported in other published studies [11, 33]. The second common barrier related to the MHD service is paying a delivery fee, however most community pharmacies in the UAE offer this service for free [3]. Most community pharmacies, specifically the chain pharmacy, which accounts for a large proportion of the community pharmacy sector in the UAE, provide both online pharmacies, and the free delivery service. Also, several dedicated delivery companies offer the medication delivery service in collaboration with community pharmacies with fixed delivery fees. This type of delivery is used mainly by individual or independent community pharmacies, or by people through direct contact with the delivery company. To compare with another country, for example, in Jordan, the community pharmacies that offer the MHD service apply a delivery fee on an individual basis, as the cost of the delivery service is added to the price of the medication [11]. In addition, the availability of the MHD service remains limited in Jordan, with the absence of a national legal framework regarding remote dispensing and informed delivery of medications, and limited funds to develop the service effectively [33].

Another issue, which is most critical related to using the MHD service, was delivering the wrong drug, which could cause harm to the patients when using, leading to a loss in faith in the service, and the pharmacy profession in general. However, 58.8% of participants were more worried in Jordan about this issue [11] than in the UAE (8.3% only).

The majority of participants were worried that online medication dispensing might limit communication with pharmacists and prevent patients from receiving appropriate medication guidance, which could result in patients being deprived of medication information that they should be aware of. The issue was also mentioned in other studies in Jordan and the UAE [2, 11, 33], which showed that the risk of medication errors might greatly increase without patient counseling. Moreover, this would affect the elderly and chronically ill patients, specifically with inaccuracies or misunderstandings of medication instructions.

The pharmaceutical regulations worldwide prohibit the dispensing of controlled medication without a prescription. In the UAE, it is prohibited to dispense any controlled and semi-controlled medications via the MHD service [13]. In addition, the pharmaceutical regulations prohibit dispensing prescribed medication without a prescription, but a proportion of global health practices are not able to control dispensing medication without a prescription [26]. This was common in community pharmacies in many countries, including medications for chronic diseases and antibiotics. As the patients used to refill their medications without a prescription by ordering the need using the electronic order system (mobile applications), but the order may not process without a prescription in some countries. However, they could do via phone as in the common pharmacy practice.

There are some limitations to this study. First, the MHD service is a relatively modern service in the community pharmacy, so this might make the participants have various expectations for the service, which will affect their opinions, specifically among the non-user of the service. Second, an online questionnaire, and a snowball sampling technique were used in this study. These might limit the generalizability of the findings. Furthermore, the potential for selection bias due to the nature of the electronic survey that was used in this study. To overcome these issues, the researchers used various media and methods to distribute the survey and invite people to participate, which ended up with an adequate sample size in this study. In addition, the study relied on self-reported data, which can be subject to recall bias since the MHD service users might have difficulty in accurately recalling past events and experiences, leading to potential inaccuracies in the information collected. Moreover, another limitation is that many participants were 40 years of age or younger. Thus, older adults and individuals with chronic diseases, who are more likely to take multiple medications, were not adequately represented in this study. This lack of representation may limit the generalizability of study findings to this population. In addition, a large proportion of participants appeared to have educational degrees. This may be attributed to a snowball effect of the data collection method using a survey.

## Conclusion

A shift in patient behavior towards adopting the MHD service is becoming an increasingly common practice in the UAE. The study provided insights into people’s perceptions, utilization patterns, and preferences of the MHD service. The results indicated the importance of the service in meeting participants’ needs in general. They particularly emphasized those seeking convenience, minimizing exposure during the pandemic, and accessibility for vulnerable populations like the elderly and children. The findings also indicated factors affecting service adoption, like quality of service, including communication between patients and pharmacists, and problems people faced, such as receiving orders late or receiving wrong orders.

As the demand for convenient healthcare services continues to grow, the findings of this study provide important insights for policymakers, healthcare providers, and pharmacies to improve the MHD service’s effectiveness and the user experience. By recognizing the strengths and weaknesses of the MHD service provided by the community pharmacies, the healthcare providers and stakeholders can develop appropriate strategies to maximize its benefits while mitigating its limitations. Efforts should be directed towards improving communication channels to ensure reliable and timely delivery that can minimize concerns about interaction with pharmacists and delivery delays. Furthermore, implementing strict quality control measures and providing adequate training and support to delivery personnel can help alleviate worries about medication errors and incorrect dispensing or delivery.

## Supplementary Information

Supplementary Material 1.

## Data Availability

Data is provided within supplementary information files.
